# Genome-wide transcriptional profiling identifies potential signatures in discriminating active tuberculosis from latent infection

**DOI:** 10.18632/oncotarget.22889

**Published:** 2017-12-04

**Authors:** Liping Pan, Na Wei, Hongyan Jia, Mengqiu Gao, Xiaoyou Chen, Rongrong Wei, Qi Sun, Shuxiang Gu, Boping Du, Aiying Xing, Zongde Zhang

**Affiliations:** ^1^ Beijing Chest Hospital, Capital Medical University, Beijing Key Laboratory for Drug Resistant Tuberculosis Research, Beijing Tuberculosis and Thoracic Tumor Research Institute, Beijing 101149, China; ^2^ Medical Laboratory, Linyi Chest Hospital, Linyi 276000, China; ^3^ Tuberculosis Department, Beijing Chest Hospital, Capital Medical University, Beijing Tuberculosis and Thoracic Tumor Research Institute, Beijing 101149, China

**Keywords:** genome-wide transcriptional profiling, signature, tuberculosis, latent tuberculosis infection

## Abstract

To better understand the host immune response involved in the progression from latent tuberculosis infection (LTBI) to active tuberculosis (TB) and identify the potential signatures for discriminating TB from LTBI, we performed a genome-wide transcriptional profile of Mycobacterium tuberculosis (M.TB)–specific antigens-stimulated peripheral blood mononuclear cells (PBMCs) from patients with TB, LTBI individuals and healthy controls (HCs). A total of 209 and 234 differentially expressed genes were detected in TB vs. LTBI and TB vs. HCs, respectively. Nineteen differentially expressed genes with top fold change between TB and the other 2 groups were validated using quantitative real-time PCR (qPCR), and showed 94.7% consistent expression pattern with microarray test. Six genes were selected for further validation in an independent sample set of 230 samples. Expression of the resistin (RETN) and kallikrein 1 (KLK1) genes showed the greatest difference between the TB and LTBI or HC groups (*P* < 0.0001). Receiver operating characteristic curve (ROC) analysis showed that the areas under the curve (AUC) for RETN and KLK1 were 0.844 (0.783–0.904) and 0.833 (0.769–0.897), respectively, when discriminating TB from LTBI. The combination of these two genes achieved the best discriminative capacity [AUC = 0.916 (0.872–0.961)], with a sensitivity of 71.2% (58.7%–81.7%) and a specificity of 93.6% (85.7%–97.9%). Our results provide a new potentially diagnostic signature for discriminating TB and LTBI and have important implications for better understanding the pathogenesis involved in the transition from latent infection to TB activation.

## INTRODUCTION

Although great effort has been made to improve the diagnosis and treatment of tuberculosis (TB), mortality and morbidity remains high, with more than 9.0 million new cases and more than 1.5 million deaths worldwide each year [1–3]. *Mycobacterium tuberculosis* (*M.TB*) infection generally remains asymptomatic as a so called latent tuberculosis infection (LTBI). However, it will reactivate to active TB (5–15%) when individuals are co-infected with HIV, are using immunosuppressive drugs, have diabetes mellitus and/or a compromised immune system [4–7]. Although host-pathogen interactions determine the outcome of *M.TB* infection, the immunological factors involved in the host immune response network in *M.TB* infection and LTBI reactivation have not yet been clearly elucidated. Up to now, there is has not been a useful method for discriminating active TB from LTBI.

Current interferon-γ release assays (IGRAs) that detect IFN-γ release from T cells in response to 2 *M.TB*–specific antigens (ESAT-6 and CFP-10), including T-SPOT.*TB* and QuantiFERON-TB, can accurately determine the presence of *M.TB* infection [8]. However, they cannot further discriminate active TB from LTBI or predict when LTBI will develop into active TB. Previous studies have demonstrated that cytokine and chemokine responses after ESAT-6 and CFP-10 stimulation have the potential to discriminate active TB from LTBI [9–11]. But these studies were limited in their scope in regards to cytokine and chemokine families and ignored the other genes that could have better discriminative potential.

Microarray platforms are capable of reliably and reproducibly measuring the expression of over 40,000 mRNA transcripts, which can encompass all of the known functional human genome [12]. A number of studies using this approach have identified different panels of diagnostic signatures that discriminate active TB from LTBI, including the genes related to IFN signaling, toll-like receptor (TLR), T- and B- cell function, TREM1 signaling and myeloid cell inflammation, *et al* [13–18]. However, these studies screened for signatures in whole peripheral blood or peripheral blood mononuclear cells (PBMCs) without *M.TB*-specific stimulation. Because many kinds of immune cells with different gene expression patterns are included in whole blood, the interpretation of blood-derived transcriptional signatures must be made in the context of the cellular composition, which was varied depending on the ethnic background and the scale of host response to a disease [19–21]. PBMCs have been used to control this variable. But without *M.TB*-specific stimulation, TB-associated transcriptional profiles may be masked in PBMCs by interference from other conditions, such as other diseases or drug use. Furthermore, the discrepancies among these studies may also be attributable to differences in genetic background.

In this study, we aimed to further uncover the host immune factors involved in the progression from LTBI to active TB and identify the potential signatures for discriminating TB from LTBI. Using PBMCs from active TB patients, LTBI individuals and healthy controls (HCs), we performed a genome-wide transcriptional profile analysis of PBMCs stimulated with the *M.TB*–specific antigens, ESAT-6/CFP-10, from which we were able to identify a TB-associated gene profile.

Our results may not only help to identify the immunological factors that may be relevant to the pathogenesis of active TB, but also provide 2 novel genes that may have the potential ability to discriminate active TB from LTBI.

## RESULTS

### Gene profiling of *M.TB-*specific antigen-stimulated PBMCs from active TB, LTBI and HCs

**Figure 1:**
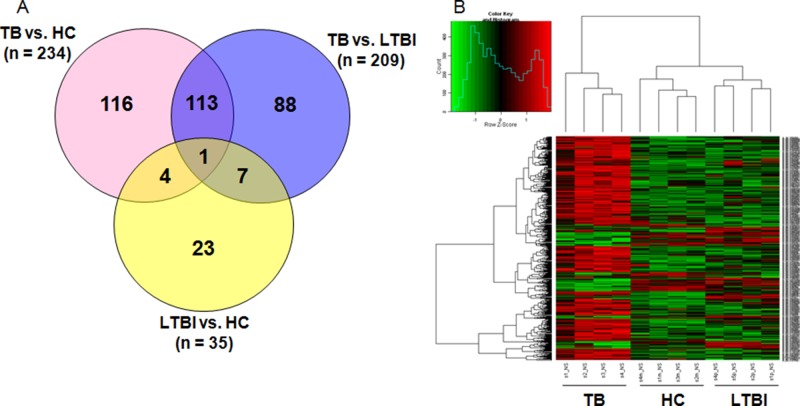
Genome-wide gene profile of M.TB-specific antigen-stimulated PBMCs from active TB, LTBI and HCs (**A**) Differentially expressed genes with *P*-value < 0.05 and fold change > 4 in pair-wise comparisons; (**B**) Unsupervised cluster analysis of 352 differentially expressed genes in the pair-wise comparisons.Note: TB, tuberculosis; LTBI, latent tuberculosis infection; HCs, healthy controls.

Genome-wide microarray analysis was employed to examine the gene expression profiles of *M.TB-*specific antigen-stimulated PBMCs from active TB, LTBI and HC groups. The genes that exhibited significant changes in expression (fold change ≥ 4 and *P*-values < 0.05 between 2 groups) were selected for further analysis (Figure 1A). Comparison of transcriptional profiles indicated that LTBI and HC groups exhibited similar patterns, with only 35 differentially expressed genes. The TB group exhibited a significantly different profile when compared to LTBI (*n* = 209) and HC (*n* = 234) groups. A total of 352 differentially expressed genes were identified when deducting the number of shared genes amongst the 3 pair-wise comparisons. Comparison of TB with the other 2 groups shared the biggest number of differentially expressed genes (*n* = 114). *F3* was the one gene that was shared in all 3 pair-wise comparisons. Unsupervised cluster analysis was performed to determine whether the 352-gene profile could reflect the different statuses of *M.TB* infection (Figure 1B). It showed that the 12 subjects were successfully clustered into 3 groups, and each group matched exactly to the clinical grouping of active TB, LTBI and HCs.

The criteria with fold change ≥ 2 and *P*-values < 0.05 between 2 groups was also used to select the differentially expressed genes, and it exhibited more differences in each pair-wise comparison (TB vs. LTBI: *n* = 1050, TB vs. HCs: *n* = 1256, LTBI vs. HCs: *n* = 479, respectively. Supplementary Figure 1).

### Bioinformatics analysis

KEGG and GO analysis was performed to further elucidate the biological functions of the differentially expressed genes in the *M.TB*-specific antigen-stimulated PBMCs between TB and the other 2 groups (LTBI and HCs). Of the 209 differentially expressed genes between TB and LTBI, the KEGG pathway analysis screened 6 statistically significant pathways (Bonferroni-corrected *P* < 0.05), while the 234 differentially expressed genes between TB and HCs were annotated to include 2 statistically significantly pathways (Bonferroni-corrected *P* < 0.05). Cytokine-cytokine receptor interaction and linoleic acid metabolism were the shared significant pathways between TB and the other 2 groups. Furthermore, the cytokine-cytokine receptor interaction pathway pooled the largest number of genes and presented the most significant difference between TB and the other 2 groups (TB vs. LTBI: included 14 genes and Bonferroni-corrected *P* = 4.394e-04; TB vs. HCs: included 18 genes and Bonferroni-corrected *P* = 4.901e-05) (Supplementary Table 1). With regard to the GO analysis, enrichment *P*-value (Bonferroni-corrected *P* < 0.001) and the pooled number of genes (included > 20 genes) were used to rank the GO term. A total of 17 ranked GO terms were detected in the comparison of TB with LTBI, while 24 ranked GO terms were detected in the comparison of TB with HCs. Furthermore, all these GO terms were associated with biological processes, including in response to stimuli, cell movement, immune response, inflammatory response and defense response (Supplementary Table 2).

### Validation of differentially expressed genes in an independent sample set

In order to validate the microarray results, the differentially expressed genes with higher expression levels (mean normalized values in the microarray test > 10) and top fold changes (> 10) between TB and the other 2 groups were selected to perform qPCR analysis using the same samples in the microarray test. Based on this criterion, 13 differentially expressed genes between TB and LTBI, and 13 differentially expressed genes between TB and HCs were selected (Table 1). Seven genes overlapped, and these genes were all statistically significant in both TB vs. LTBI and TB vs. HCs in microarray test comparisons (*P* < 0.05). All 19 genes were confirmed by qPCR analysis using the samples from the microarray test, and 18 genes (94.7%) showed consistent expression patterns with the microarray results.

**Table 1:** Differentially expressed genes with fold change >10 and higher expression level in the comparison of TB with LTBI and HC groups

Genes	Microarray test			qPCR validation		
	Fold Change	Regulation	*P*-value	Fold Change	Regulation	*P*-value
**TB/LTBI**						
RETN	19.71	Up	0.006	17.79	Up	0.087
CXCL5	20.46	Up	0.013	31.07	Up	0.031
HP	14.23	Up	0.008	4.99	Up	0.038
LCN2	13.24	Up	0.007	9.53	Up	0.085
S100A12	12.71	Up	0.001	5.33	Up	0.101
ABCA1	59.84	Up	0.0002	22.96	Up	0.148
PID1	17.68	Up	0.003	9.61	Up	0.003
LTF	16.56	Up	0.008	3.99	Up	0.296
LRRC38	15.57	Up	0.003	2.51	Up	0.372
MT1JP	15.16	Up	0.007	8.39	Up	0.235
CD177	12.59	Up	0.002	1.74	Up	0.235
CXCL3	11.20	Up	0.001	7.61	Up	0.036
INSM1	10.26	Up	0.004	4.26	Up	0.050
**TB/HCs**						
RETN	16.02	Up	0.011	15.04	Up	0.048
KLK1	0.09	Down	0.046	0.07	Down	0.036
CXCL5	12.29	Up	0.007	6.35	Up	0.023
HP	11.65	Up	0.016	2.88	Up	0.071
F3	22.30	Up	0.005	19.98	Up	0.163
CYP3A5	16.54	Up	0.001	6.18	Up	0.011
CNKSR3	11.51	Up	0.006	6.68	Up	0.218
CYP3A7	10.89	Up	0.002	0.96	Down	0.862
CA12	10.10	Up	0.015	3.79	Up	0.155
ABCA1	15.13	Up	0.012	4.16	Up	0.185
LRRC38	20.95	Up	0.002	3.20	Up	0.233
MT1JP	14.96	Up	0.007	9.94	Up	0.201
CD177	15.93	Up	0.001	3.31	Up	0.063

Note: TB, tuberculosis; LTBI, latent tuberculosis infection; HCs, healthy controls.

**Figure 2:**
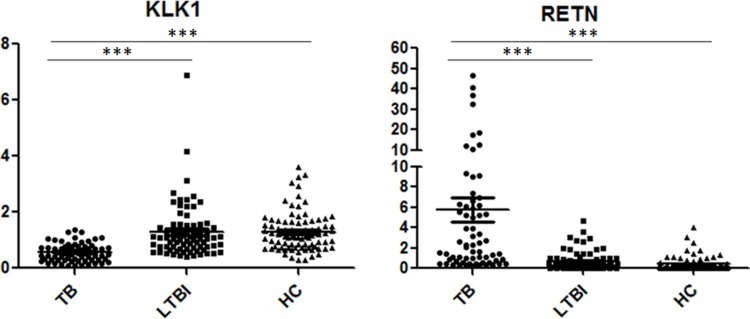
Scatter plots of RETN and KLK1 gene expression values in comparison of TB with LTBI and HCs by qPCR Note: TB, tuberculosis; LTBI, latent tuberculosis infection; HCs, healthy controls. ^***^P < 0.0001; Horizontal bar, the relative expression value of validated genes. The mRNA values of the validated genes were normalized to the housekeeping gene *GAPDH*. The numbers of participants in validation test were the following: TB, *n* = 66; LTBI, *n* = 78; HCs, *n* = 86.

The 6 genes (RETN, KLK1, CXCL5, HP, PID1 and CXCL3) that showed statistically significant differences in qPCR analysis (TB vs. LTBI, TB vs. HCs, *P* < 0.05) were further validated in an additional independent sample set, which included 66 TB patients, 78 LTBI individuals and 86 HCs (Table 2). Three genes (RETN, KLK1 and HP) displayed significantly different expression between TB and the other 2 groups, and the regulation patterns were consistent with the microarray study. Among these genes, RETN and KLK1 exhibited a statistically significant difference (*P* < 0.0001 and fold change > 2) in the comparisons of TB vs. LTBI and TB vs. HCs. Scatter plots of these 2 genes are shown in Figure 2.

**Table 2:** Validation of the differentially expressed genes in an independent sample set

Genes	TB/LTBI		TB/HCs	
	Fold Change	*P*-value	Fold Change	*P*-value
RETN	8.59	5.22E-05	13.85	2.35E-05
KLK1	0.44	9.18E-09	0.43	1.17E-013
CXCL5	0.40	0.007	0.51	0.009
HP	2.18	0.035	2.16	0.040
PID1	0.66	0.320	1.48	0.164
CXCL3	0.10	0.075	0.23	0.108

Note: TB, tuberculosis; LTBI, latent tuberculosis infection; HCs, healthy controls.

The independent sample set included 66 TB patients, 78 LTBI individuals and 86 HCs.

### RETN and KLK1 to distinguish TB from LTBI and HCs

**Figure 3:**
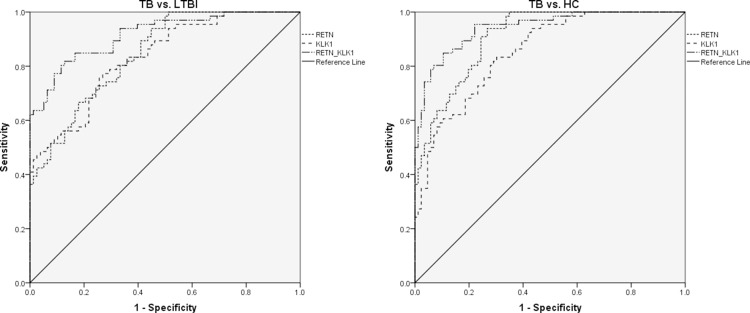
The discriminative performance of RETN and KLK1 genes in discriminating TB from LTBI and HCs by receiver operating characteristic curve (ROC) analysis Note: TB, tuberculosis; LTBI, latent tuberculosis infection; HCs, healthy controls. The numbers of participants were the following: TB, *n* = 66; LTBI, *n* = 78; HCs, *n* = 86. The receiver operating characteristic curve depicts the sensitivity and specificity of the potential signatures in discriminating TB from LTBI and HCs. The area under the ROC curve (AUC) in discriminating TB from LTBI: RETN, 0.844 (0.783–0.904); KLK1, 0.833 (0.769–0.897); Combination of RETN and KLK1, 0.916 (0.872–0.961). The AUCs in discriminating TB from HCs: RETN, 0.908 (0.864–0.951); KLK1, 0.853 (0.796–0.911); Combination of RETN and KLK1, 0.943 (0.908–0.977).

Due to the significantly different expression of RETN and KLK1 genes between TB and the other 2 groups, we attempted to detect whether these 2 genes or a combination thereof could discriminate TB from LTBI and HCs. A receiver operating characteristic curve (ROC) analysis was performed to evaluate the discriminative potential of these 2 genes in the validation set (Figure 3 and Table 3). The areas under the curve (AUCs) of RETN and KLK1 were 0.844 (0.783–0.904) and 0.833 (0.769–0.897), respectively, in discriminating TB from the LTBI group, and the AUC values of RETN and KLK1 were 0.908 (0.864–0.951) and 0.853 (0.796–0.911), respectively, in discriminating TB from the HC group. Logistic regression with forward stepwise analysis indicated that RETN and KLK1 were included in the diagnostic model in discriminating TB from the other 2 groups. The AUC values of this combination could reach as high as 0.916 (0.872–0.961) in discriminating TB from LTBI with a sensitivity of 71.2% (58.7%–81.7%) and a specificity of 93.6% (85.7%–97.9%). In regards to discriminating TB from HCs, AUC values of this combination could reach as high as 0.943 (0.908–0.977) with a sensitivity of 84.9% (73.9%–92.5%) and a specificity of 89.5% (81.1%–95.1%). These results revealed that the combination of these genes could achieve better discriminative capacity than any single gene.

**Table 3:** ROC analysis of RETN and KLK1 genes for discriminating TB from LTBI and HC groups

Signatures	AUC (95% CI)	Sensitivity (95% CI)	Specificity (95% CI)
**TB vs. LTBI**			
RETN	0.844(0.783–0.904)	74.2 (62.0–84.2)	71.8 (60.5–81.4)
KLK1	0.833 (0.769–0.897)	75.8 (63.6–85.5)	74.4 (63.2–83.6)
RETN-KLK1 Combination	0.916 (0.872–0.961)	71.2 (58.7–81.7)	93.6 (85.7–97.9)
**TB vs. HCs**			
RETN	0.908 (0.864–0.951)	93.9 (85.2– 98.3)	73.3 (62.6–82.2)
KLK1	0.853 (0.796–0.911)	81.8 (70.4– 90.2)	70.9 (60.1–80.2)
RETN-KLK1 Combination	0.943 (0.908–0.977)	84.9 (73.9–92.5)	89.5 (81.1– 95.1)

Note: TB, tuberculosis; LTBI, latent tuberculosis infection; HCs, healthy controls;

ROC, receiver operating characteristic curve; AUC, the area under the ROC curve.

## DISCUSSION

The outcome of *M.TB* infection (active TB or LTBI) is based on the interaction between *M.TB* and host immunity. Identifying the immunologic parameters involved in active TB will help improve early diagnosis and facilitate our understanding of the pathogenesis involved in the transition of latent infection to TB activation. In this study, microarray techniques were applied to compare the transcriptional profiles of active TB patients, LTBI and HC individuals. Further qPCR analyses validated our microarray results and confirmed that the expression of RETN and KLK1 genes was significantly different between TB and the other 2 groups (LTBI and HCs). The combination of these 2 genes demonstrated the potential to discriminate TB from LTBI and HCs.

Unlike previous microarray studies using non-stimulated whole blood or PBMCs, our study examined the transcriptional profiles of *M.TB*-specific antigen (ESAT-6/CFP-10)-stimulated PBMCs to investigate gene expression changes between TB and the other 2 groups. ESAT-6 and CFP-10 have been confirmed as specific antigens with good immunogenicity in pathogenic *M.TB* and have been widely used in the IGRA tests and novel skin tests for detecting *M.TB* infection [8, 22, 23]. Compared with tuberculin (PPD)-stimulation, which is a mixture of multiple *M.TB* antigens, stimulation with ESAT-6 and CFP-10 can induce a *M.TB*-specific immune response, while excluding the non-specific immune response triggered by other mycobacterium or BCG vaccination. Furthermore, based on IGRA tests or novel skin tests that discriminate *M.TB* infection from healthy controls, the differentially expressed genes of ESAT-6 and CFP-10-stimulated PBMCs between TB and LTBI may further discriminate the different infection status.

Similar with previous studies [24, 25], the differentially expressed genes between TB and the other 2 groups in our study were primarily associated with stimulus response, indicating the variances of PBMCs in response to *M.TB*-specific antigens between different infection statuses. KEGG analysis showed that differentially expressed genes between TB and the other 2 groups were mainly pooled in cytokine-cytokine receptor pathways, suggesting that the cytokine and cytokine receptor genes are important in TB occurrence and development. These results are consistent with previous studies that detected different patterns of cytokines or chemokines between TB and non-TB group (LTBI, HCs or other pulmonary diseases) in response to *M.TB*-specific antigens or *M.TB* strains [9–11]. Recently, a blood RNA signature, including 16 genes for tuberculosis disease risk, has been validated [26]. Two of these 16 genes were also differentially expressed between TB and LTBI in our study (APOL, *P*-value = 0.031; GBP4, *P*-value = 0.044). Kaforou et al found a 27-transcript signature distinguished TB from LTBI using a whole blood RNA microarray [16]. Four of these 27 genes were also differentially expressed between TB and LTBI in our study (GAS6, *P*-value = 0.0076; S100A8, *P*-value = 0.0030; C5, *P*-value = 0.029; FAM20A, *P*-value = 0.028.). Among the 393-gene signature of active TB from Berry et al [15], 32 genes also presented a significant difference between TB and the other 2 groups in our study. The overlap of genes between previous studies and our study indicates that our microarray test results are reliable.

For the first time, RETN was detected as a differentially expressed gene that is increased in active TB patients compared to LTBI and HC individuals upon stimulation with *M.TB*-specific antigens. RETN is a 12.5 kDa cysteine-rich peptide that is mainly produced by monocytes and macrophages [27]. As a member of the resistin-like molecule (RELM) family, a small family of secreted pro-inflammatory proteins, RETN can trigger a pro-inflammatory state in inflammatory responses, mediates metabolic disturbances and promotes cell proliferation [28, 29]. Previous studies have suggested that RETN is associated with type 2 diabetes [30, 31], which indicates that RETN could be involved in the host immune response process. It has been reported that RETN can induce production of pro-inflammatory cytokines and chemokines in PBMCs and, can promote macrophage polarization in an independent process [32–34]. In our study, we found that RETN expression was significantly increased in active TB patients compared to LTBI and HC individuals. The higher expression of RETN may induce the increased pro-inflammatory cytokines production, which were also detected in TB patients in our study.

Human tissue kallikreins (KLKs) are a 15-member group of serine proteases. KLK1 is a key component of the kallikrein-kinin system and may activate protease activated receptors (PARs) in inflammatory and cardiovascular diseases [35, 36]. It is well established that KLK1 can prompt Lysyl-bradykinin release from low molecular weight kininogen through the cleavage of 2 peptide bonds involving Met-Lys and Arg-Ser, which confers to KLK1 the ability to act as both a chymotrypsin and a trypsin-like enzyme [37]. Furthermore, it has been demonstrated that KLK1 can protect against lupus and anti-glomerular basement membrane-specific antibody-induced nephritis in mice and humans [38], indicating KLK1 may be involved in autoimmunity response processes. In our study, KLK1 gene expression was decreased in active TB patients. Although there is no report on the association between KLK1 and active TB, recent microarray analyses have also detected that the expression of KLK11 and KLK12 are decreased in monocyte-derived macrophages (MDMs) from *Mycobacterium bovis*-infected cows [39].

Although the RETN or KLK1 genes alone have moderate performance in discriminating active TB from LTBI and HCs, their discriminative ability was enhanced when these 2 genes were combined. The immunological changes that occur when transitioning from a healthy status to *M.TB* infection and then to active TB are undoubtedly comprehensive and complicated. Thus, it is unrealistic to rely on one single gene to accurately diagnose active TB. The combination of multiple genes will substantially achieve better diagnostic performance [40]. Previous studies on discovering the biomarkers for active TB diagnosis have also detected a panel of genes that have better discriminative ability [15, 16, 26].

To our knowledge, this is the first study using *M.TB*-specific antigen (ESAT-6/CFP-10)-stimulated PBMCs to elucidate the differences of human immune responses between active TB, LTBI and healthy controls. Notably, our study was performed in a Chinese population, whereas previous studies were performed in European and South African populations that have different genetic and TB epidemiology backgrounds. However, there are some limitations in our study. First, due to the small number of the samples in the microarray set, the effect of inter-individual differences cannot be avoided, although independent sample sets were used to validate the microarray results. Second, although the number of differentially expressed genes between TB and the other groups in the microarray test were numerous, only the genes with top fold change were selected for validation by qPCR. We cannot exclude the genes with moderate fold change may also provide discriminative value. Third, there is lack of controls for non-TB pulmonary diseases. Further studies with well-designed patient and control groups are required to validate the usefulness of the identified genes for TB. Lastly, a blinded sample set was not used when validating the discriminative ability of RETN and KLK1. A further study utilizing a larger sample size and blinded analyses should be performed to validate the combination of RETN and KLK1.

In conclusion, our study uncovered a genome-wide expression profile of *M.TB*-specific antigen-stimulated PBMCs in different *M.TB* infection statuses and identified the combination of RETN and KLK1 genes to have potential value in distinguishing active TB from LTBI individuals and healthy controls. These results provide a new potential diagnostic signature for discriminating active TB and LTBI and have important implications for better understanding the pathogenesis involved in the transition from latent infection to TB activation.

## MATERIALS AND METHODS

### Subjects

Active TB patients were recruited from Beijing Chest Hospital between May 2009 and September 2017. All TB patients were recruited with typical TB clinical symptoms, chest radiograph revealing TB lesion, at least 2 consecutive positive sputum smears or a positive sputum culture. They had not received anti-TB treatment within the past 30 days. LTBI individuals and healthy controls were recruited from TB screening campaigns, which were conducted in Beijing between November 2009 and September 2017. LTBI individuals satisfied the following criteria: positive tuberculin skin test (TST) and T-SPOT.*TB* results, normal chest radiograph, and without any clinical evidence of active TB and other diseases. Healthy controls were people with negative TST and T-SPOT.TB tests, normal chest radiograph and no clinical symptoms of diseases. Individuals with positive human immunodeficiency virus (HIV), positive hepatitis B virus (HBV) or hepatitis C virus (HCV), diabetes, malignancies, severe autoimmune diseases, and those who took immunosuppressive or immunopotentiator agents, or were in pregnancy or lactation were excluded.

In the microarray study, 12 subjects were enrolled within 3 groups: active TB (*n* = 4), LTBI (*n* = 4), and HCs (*n* = 4). Additional 66 active TB patients, 78 LTBI individuals, and 86 HCs were enrolled for further qPCR validation. The demographic characteristics of all 242 participants in this study are shown in Table 4. This study was performed in accordance with the guidelines of the Helsinki Declaration and was approved by the Ethics Committee of the Beijing Chest Hospital, Capital Medical University. Written informed consents were obtained from each participant before blood sample collection.

**Table 4:** Demographic characteristics of the study participants

Study complex	Characters	TB	LTBI	HCs
Microarray set	n	4	4	4
	Male/female	1/3	0/4	0/4
	Age (median, range)	35 (33–39)	36 (32–42)	38 (33–44)
	BMI (mean ± SD)	23.3 ± 0.8	23.6 ± 3.4	23.6 ± 3.6
	Smokers/non-smokers	0/4	0/4	0/4
	BCG vaccination, *n* (%)	4 (100)	4 (100)	4(100)
Validation set	n	66	78	86
	Male/female	40/26	48/30	47/39
	Age (median, range)	35 (18–70)	38 (23–61)	34 (19–60)
	BMI (mean ± SD)	20.6 ± 3.1	23.6 ± 3.1	23.3 ± 3.3
	Smokers/non-smokers	23/43	18/60	19/67
	BCG vaccination, *n* (%)	64 (97.0)	73 (93.6)	81 (94.2)

Note: TB, tuberculosis; LTBI, latent tuberculosis infection; HCs, healthy controls;

n, number of subjects; SD, standard deviation; BMI, body mass index.

### PBMCs isolation and *in vitro* stimulation

Peripheral blood samples (6ml) were collected in heparin-containing vacutainer tubes from each subject. PBMCs were separated by density gradient using Lympholyte Cell Separation Media (Tianjin Haoyang Biological Manufacture Co., Ltd, China) within 6 hours of blood collection. The number of live cells were counted using Muse Cell Analyzer (Merck & Millipore, Germany). PBMCs were cultured with AIM-V (Invitrogen Life Technology, USA) containing 2mM L-glutamine, 50 ug/ml streptomycin sulfate, 10 ug/ml gentamicin sulfate, and stimulated with 10 ug/ml purified *M.TB*-specific antigens ESAT-6/CFP-10 for 24 h at 37 °C in a humidified incubator with 5% CO_2_ [11].

### RNA extraction

Total RNA was extracted from PBMCs using the miRneasy^®^ Mini kit (QIAGEN, Germany) according to the protocols recommended by the manufacturer. RNase-free DNase I (QIAGEN, Germany) was used to remove the genomic DNA contamination. The integrity and quality of RNA was evaluated using an Agilent 2100 Bioanalyzer (Agilent Technology). RNA with a 2100 RIN (RNA integrity number) ≥ 7.0 and 28S/18S > 0.7 was used for the microarray study and qPCR validation.

### Microarray test and bioinformatics analysis

RNA samples from each group were used to generate fluorescence labeled cRNA targets for the Agilent Whole Human Genome Oligo Microarray (4 × 44 K, including ~41,000 genes and transcripts). Labeled cRNA targets were then hybridized with the slides. After hybridization, slides were scanned on the Agilent Microarray Scanner (Agilent technologies, Santa Clara, CA, USA). Data were extracted with Feature Extraction software 10.7 (Agilent technologies, Santa Clara, CA, USA). Raw data were normalized by Quantile algorithm, GeneSpring Software 12.6.1 (Agilent technologies, Santa Clara, CA, US). The microarray experiments were performed by following the protocol of Agilent technologies Inc. at Shanghai Biotechnology Corporation. The microarray data was deposited in GEO database: GSE98461.

Fold changes of gene expression values were calculated in 2 pair-wise comparisons. Differentially expressed genes were identified and selected for further analysis based on the *P*-value < 0.05 and with a fold change of at least 2 or more. The selected genes were grouped in functional categories based on Gene Ontology database (GO: http://www.geneontology.org/), and functional pathways(KEGG) were also analyzed by using online SAS analysis system.

### Quantitative real-time PCR analysis

A total of 200 ng of purified RNA was reverse transcribed to cDNA using SuperRT cDNA Synthesis Kit (CWbio.Co., Ltd, China) according to the manufacturer's protocol. SYBR Green (Power SYBR Green PCR Master Mix, Applied Biosystems, Inc.) uptake in double-stranded DNA was measured using the ABI 7900 Real-time PCR System (Applied Biosystems, Inc.). We calculated 2^-ΔΔCT^ and used this statistic to determine relative gene expression. The reference gene was GAPDH. The primer sequences of the target genes in the qPCR analysis are shown in Supplementary Table 3.

### Data analysis

The differentially expressed genes between the 2 groups were analyzed using the *t*-test. The receiver operating characteristic curve (ROC) analysis was performed to determine the discriminative ability of selected genes to distinguish TB from LTBI and HCs, with the overall accuracy assessed by the area under the ROC curve (AUC) values. Significance was inferred for *P* < 0.05. All statistical analysis was performed using the commercial statistical software SPSS version 21.0 (SPSS, Inc., Chicago, IL, USA).

## SUPPLEMENTARY MATERIALS FIGURES AND TABLES


