# Supplementary material for: Genome-wide transcriptional profiling identifies potential signatures in discriminating active tuberculosis from latent infection
Source: Oncotarget. 2017 Dec 4;8(68):112907–16. doi: 10.18632/oncotarget.22889 (PMC5762561; doi:10.18632/oncotarget.22889)
Supplement: Supplementary file 1 [file oncotarget-08-112907-s001.pdf]

Genome-wide transcriptional profiling identifies potential signatures in discriminating active tuberculosis from latent infection

SUPPLEMENTARY MATERIALS

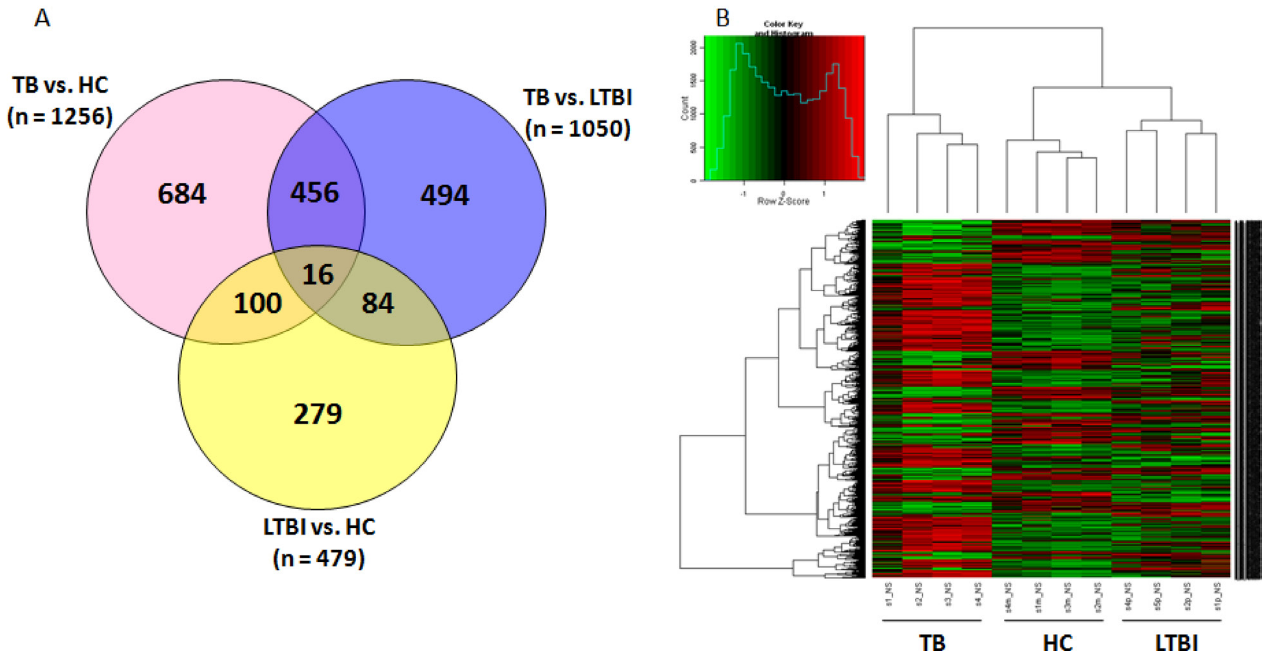

Supplementary Figure 1: Differentially expressed genes of M.TB-specific antigens stimulated PBMCs from active TB, LTBI and HCs with *P*-value < 0.05 and fold change > 2 in pair-wise comparisons (**A**) and the unsupervised cluster analysis of 2113 differentially expressed genes in the pair-wise comparisons (**B**). Note: TB, tuberculosis; LTBI, latent tuberculosis infection; HC, healthy control.

Supplementary Table 1: KEGG analysis of the differentially expressed genes between TB and the other two groups

Pathway ID	Pathway Term	Count	Percent (%)	*P*-value	Bonferroni-corrected *P*-value
**TB vs. LTBI**					
hsa04060	Cytokine-cytokine receptor interaction	14	20.59	4.83E-06	4.39E-04
hsa05143	African trypanosomiasis	4	5.88	1.54E-04	7.02E-03
hsa00591	Linoleic acid metabolism	3	4.41	1.03E-03	2.34E-02
hsa05144	Malaria	4	5.88	8.60E-04	2.61E-02
hsa05323	Rheumatoid arthritis	5	7.35	2.00E-03	3.64E-02
hsa05412	Arrhythmogenic right ventricular cardiomyopathy (ARVC)	4	5.88	4.38E-03	4.98E-02
**TB vs. HC**					
hsa04060	Cytokine-cytokine receptor interaction	18	20.00	4.19E-07	4.90E-05
hsa00591	Linoleic acid metabolism	4	4.44	2.64E-04	1.55E-02

Note: KEGG pathways with Bonferroni *P*-values < 0.05 were presented.

Supplementary Table 2: GO analysis of the differentially expressed genes between TB and the other two groups

GO Accession	GO Term	Gene Count	Percent (%)	*P*-value	Bonferroni-corrected *P*-value
**TB vs. LTBI**					
GO:0016477	cell migration	47	24.61	1.37E-14	4.51E-11
GO:0048870	cell motility	47	24.61	2.68E-13	4.42E-10
GO:0040011	locomotion	56	29.32	1.44E-12	1.59E-09
GO:0009617	response to bacterium	27	14.14	1.94E-11	1.60E-08
GO:0051707	response to other organism	34	17.80	2.44E-10	1.01E-07
GO:0006928	cellular component movement	53	27.75	3.70E-10	1.35E-07
GO:0009607	response to biotic stimulus	34	17.80	6.38E-10	2.10E-07
GO:0030334	regulation of cell migration	24	12.57	5.17E-08	1.31E-05
GO:0040012	regulation of locomotion	26	13.61	5.57E-08	1.31E-05
GO:0033993	response to lipid	27	14.14	1.12E-07	2.17E-05
GO:0009605	response to external stimulus	56	29.32	1.11E-07	2.28E-05
GO:2000145	regulation of cell motility	24	12.57	1.48E-07	2.57E-05
GO:0006954	inflammatory response	23	12.04	2.10E-07	3.47E-05
GO:0009611	response to wounding	35	18.32	6.10E-07	8.37E-05
GO:0051270	regulation of cellular component movement	24	12.57	8.92E-07	1.18E-04
GO:0006952	defense response	43	22.51	1.01E-06	1.28E-04
GO:0006955	immune response	41	21.47	1.17E-06	1.43E-04
**TB vs. HC**					
GO:0032496	response to lipopolysaccharide	23	10.60	2.30E-12	3.94E-09
GO:0002237	response to molecule of bacterial origin	23	10.60	4.98E-12	5.71E-09
GO:0016477	cell migration	45	20.74	1.43E-11	1.23E-08
GO:0040011	locomotion	58	26.73	2.25E-11	1.55E-08
GO:0009611	response to wounding	47	21.66	5.30E-11	3.03E-08
GO:0048870	cell motility	46	21.20	6.64E-11	3.26E-08
GO:0009617	response to bacterium	26	11.98	1.47E-09	5.60E-07
GO:0009607	response to biotic stimulus	36	16.59	1.41E-09	6.05E-07
GO:0006954	inflammatory response	28	12.90	3.02E-09	1.04E-06
GO:0006928	cellular component movement	55	25.35	4.23E-09	1.32E-06
GO:0051707	response to other organism	34	15.67	6.11E-09	1.75E-06
GO:0009605	response to external stimulus	63	29.03	2.82E-08	7.45E-06
GO:0006952	defense response	51	23.50	3.12E-08	7.65E-06
GO:0030334	regulation of cell migration	26	11.98	4.16E-08	8.93E-06
GO:0040012	regulation of locomotion	28	12.90	5.81E-08	1.11E-05
GO:0051270	regulation of cellular component movement	28	12.90	7.70E-08	1.39E-05
GO:2000145	regulation of cell motility	26	11.98	1.27E-07	2.18E-05
GO:0033993	response to lipid	29	13.36	1.36E-07	2.22E-05
GO:0006955	immune response	44	20.28	2.23E-06	2.25E-04
GO:0009888	tissue development	42	19.35	4.40E-06	4.20E-04
GO:0042127	regulation of cell proliferation	39	17.97	4.66E-06	4.33E-04
GO:0042060	wound healing	24	11.06	6.42E-06	5.66E-04
GO:0002376	immune system process	58	26.73	6.93E-06	5.95E-04
GO:0008283	cell proliferation	46	21.20	8.13E-06	6.49E-04

Note: GO terms with Bonferroni *P*-values < 0.001 and including > 20 genes were presented.

Supplementary Table 3: Target genes and primer sequences

Genes	Forward primer (5′to3′)	Reverse primer (5′to3′)
CXCL5	TTGGCCCCTTTCACAGAGTAGA	GAACTGTGCTAAAAACCCGACA
CYP3A5	TGGACAGAGCCTGAGGAGTT	GTTTCTGGGTCCAGTTCCAA
HP	AGCCTGGAAGAGGGCAAAGT	CCCATCAGCTTCAAACCACAT
LCN2	GGGCCTCCCTGAAAACCA	TGCACTCAGCCGTCGATACA
S100A12	GGTAGCCATTGCGCTGAAG	AGGACATTGCTGGGTAAAAAGC
CD177	CAGACCACCGTTCTATACGCAAT	CCCACGATACGCAGATGCT
F3	AAGGTGACTGGGAATTGTTACTG	GATACGTTGTTGTAAGCCACTGA
CXCL3	TAAATGACAGGGTGGGGAAC	CCCTTACCCTAACAGTGATCCA
LRRC38	TGGGATTAGTGCCCTCATGAA	GCTGCATCCTCAAGCATTGA
ABCA1	TTTTTGTGCTCTTTGTTCATCATTG	CCAGTGCAAAAATAGATCCCATT
CA12	GCCCACACGCTCCTAACTCT	CCTGGGCCTTGTTTTTGCTA
CNKSR3	TTGCATTTTGACCTGTTCAGTGT	CCATGAGGCTTTCCAAGATATTTC
CYP3A7	GGCTTCTCTGCTTCTCATAGGACTA	TTATGTTATCAGAGCTCAGGAGGAGTT
INSM1	CTGTTGTCTGGGATTGTTTTGTG	ACGTGAAACACTGAGGCAGTTACT
KLK1	CACCCCCAATAAGCCTTCTG	TCCGCTATGGTGTCCTCGAT
MT1JP	GCAAAGGGACGTCGGAGAA	TCCAGGTTGTGCAGGTTGTTC
PID1	GTGTCCGTATCTGCGTTTGTGT	CATGCTTATTCTACATGCCTGAAAA
RETN	TCACCGGCTGCACTTGT	CTGGCAGTGACATGTGGTCT
LTF	TCACTGCCCCCAGCTCTTC	TAAGCAGATGGATGGGCAATC
GAPDH	TGACTTCAACAGCGACACCCA	CACCCTGTTGCTGTAGCCAAA

Note: GAPDH was a housekeeping gene.
